# Interpretable Machine Learning for Predicting Adverse Pregnancy Outcomes in Gestational Diabetes: Retrospective Cohort Study

**DOI:** 10.2196/71539

**Published:** 2025-09-16

**Authors:** Jiaxi Li, Xiali Liu, Shenyang He, Yan Ren

**Affiliations:** 1 Jinniu Maternity and Child Health Hospital of Chengdu, Chengdu, China; 2 Department of Anesthesiology, West China Hospital, Sichuan University, Chengdu, China; 3 Department of Endocrinology and Metabolism, Adrenal Center, West China Hospital of Sichuan University, Number 37 Guoxue Alley, Wuhou District, Chengdu, 610041, China, 86 18227387870

**Keywords:** gestational diabetes mellitus, adverse pregnancy outcomes, interpretable model, machine learning, risk prediction, ensemble learning

## Abstract

**Background:**

Gestational diabetes mellitus (GDM) affects over 5% of pregnancies worldwide, elevating risks of type 2 diabetes post partum and complications such as fetal death, miscarriage, and congenital abnormalities. Effective GDM management is essential to balance glycemic control and pregnancy outcomes.

**Objective:**

We aim to develop interpretable machine learning models using GDM datasets for predicting adverse pregnancy outcomes and identifying key factors through the Shapley additive explanations (SHAP) algorithm, thus supporting improved maternal and infant health.

**Methods:**

Data preprocessing and feature selection were performed, with adaptive synthetic sampling used to address class imbalance. Classification models, including logistic regression, random forest, support vector machine, and extreme gradient boosting, were built and enhanced through the stacking method. Model interpretability was assessed with SHAP to quantify feature contributions.

**Results:**

Among 1670 patients, 200 experienced adverse outcomes. The stacking model outperformed individual models, achieving an accuracy of 85.6%, a sensitivity of 57.8%, a specificity of 95.9%, and an area under the receiver operating characteristic curve of 0.82 on the test set. External validation on 159 patients showed a decline in performance (accuracy 83.6%, area under the receiver operating characteristic curve 0.67). SHAP analysis identified gestational age, glucose control, and diagnosis time among the most influential predictors, providing clinically meaningful insights into risk factors. Additionally, detailed SHAP-based visualization revealed the distribution of different feature values and their nonlinear impact on outcomes, as well as interaction effects between features. These interpretable analyses enabled a deeper understanding of individual and combined feature contributions, thereby enhancing clinical assessment capabilities.

**Conclusions:**

This study underscores the potential of machine learning in predicting adverse outcomes in GDM, with interpretable features offering valuable clinical insights to enhance pregnancy management and maternal-infant health.

## Introduction

Gestational diabetes mellitus (GDM) is a significant global health concern, affecting at least 5% of pregnancies worldwide [1]. It is characterized by impaired glucose tolerance due to an increase in anti-insulin substances and decreased insulin sensitivity, leading to hyperglycemia and other metabolic disturbances [2]. Women diagnosed with GDM not only face increased perinatal complications but are also at a significantly higher risk of developing type 2 diabetes mellitus postpartum [3]. Moreover, in utero exposure to hyperglycemia has long-term implications for offspring, predisposing them to metabolic disorders, obesity, and cardiovascular diseases later in life.

Uncontrolled GDM significantly increases the risk of adverse pregnancy outcomes, including fetal macrosomia, preterm birth, neonatal hypoglycemia, and even perinatal mortality [4,5]. During early pregnancy, poor glycemic control may result in congenital malformations or spontaneous abortion, whereas in later stages, it can contribute to pre-eclampsia, respiratory distress syndrome, and hyperbilirubinemia [6,7]. Despite advancements in clinical management, the timely identification of high-risk pregnancies remains a challenge. Traditional risk assessment methods, such as clinical scoring systems and conventional regression models, often rely on predefined risk factors and fail to capture complex interactions within high-dimensional clinical data [8]. This limitation highlights the need for more advanced predictive tools that can improve early risk stratification and optimize maternal and fetal outcomes.

Machine learning has emerged as a transformative approach in medical research, offering superior capabilities in analyzing complex, high-dimensional datasets [9]. Machine learning models can effectively capture intricate patterns and nonlinear relationships in clinical data, surpassing traditional statistical approaches in predictive accuracy. In a retrospective analysis of 8888 deliveries, random forest classification achieved exceptional accuracy in predicting birth asphyxia, with critical risk factors including maternal hypertension, anemia, preterm birth, and noncephalic presentation [10]. Similarly, deep learning showed superior predictive performance for intrauterine growth restriction in another study of 8888 pregnancies, identifying key risk factors such as maternal hypertension, drug addiction, and COVID-19 infection [11]. Furthermore, in an analysis of 7166 pregnancies, random forest demonstrated optimal prediction of fetal heart, with significant predictors including primiparity, placental abruption, and male fetal sex [12]. However, the use of machine learning models in predicting GDM-related adverse pregnancy outcomes remains relatively limited—particularly in the field of interpretable machine learning. Most current studies focus on model predictive accuracy, lacking transparent analysis of feature variable impacts, which makes it difficult to provide clinically reliable risk factor assessments. This lack of interpretability limits the practical application and adoption of such models in real-world medical settings. Therefore, there is an urgent need to develop models that combine high predictive performance with clinical interpretability to identify key risk factors and assist physicians in formulating personalized intervention strategies.

To address this gap, this study aims to develop an interpretable machine learning model using a publicly available GDM dataset to predict the risk of adverse pregnancy outcomes associated with gestational diabetes. Unlike previous studies that primarily focus on GDM diagnosis, our approach emphasizes predicting maternal and fetal complications, which is crucial for timely intervention. Furthermore, we incorporate Shapley additive explanations (SHAP) analysis to enhance model interpretability, allowing clinicians to identify the most influential factors contributing to adverse pregnancy outcomes. By providing clinically actionable insights, our model aims to support personalized risk assessment and improve maternal and fetal health outcomes.

## Methods

### Data

This study is based on the publicly available GDM dataset. We included pregnant women who met specific diagnostic criteria through the publicly available dataset [13,14]. The dataset comprises anonymized electronic health records extracted from the Cerner system at St Mary’s Hospital in London, covering pregnancies monitored between April 2016 and November 2019. A total of 1854 records were initially retrieved, including clinical and demographic variables such as maternal age, BMI at booking, ethnicity, glucose tolerance test results (0- and 120-min post-75 g glucose load), mode of delivery, gestational age, neonatal birth weight, and stillbirth outcomes.

To ensure data quality and reliability, patients with missing values in key variables were excluded. Implausible outliers were identified and corrected when possible; otherwise, they were removed from the dataset. Unit inconsistencies were also standardized. These preprocessing steps were applied to ensure consistency, accuracy, and completeness of the data used for model development and analysis.

### Subject

To ensure the accuracy and reliability of this study’s results, inclusion criteria (based on the results of the oral glucose tolerance test [OGTT]) were defined as follows: the blood glucose level of pregnant women should reach or exceed 7.8 mmol/L and not exceed 25 mmol/L at 2 hours after OGTT; or the blood glucose level should reach or exceed 5.6 mmol/L and not exceed 25 mmol/L at 10 minutes after OGTT [15]. In addition, the study’s definition of adverse pregnancy outcomes includes newborn birth weight below 2500 g, severe prematurity, stillbirth, and Apgar score below 4 [16]. Additionally, we excluded cases with missing Apgar score data to avoid potential biases that could arise from inaccuracies in assessing pregnancy outcomes. By implementing these strict inclusion and exclusion criteria, we ensured the consistency of this study’s population, allowing for a more accurate assessment of the impact of GDM on the health of pregnant women and their offspring.

### Data Preprocessing

In this study, we encountered issues with missing values for multiple features in the dataset. To maintain data integrity and the reliability of the analysis, we adopted a cautious approach to handle these missing values. Specifically, when the proportion of missing values for a particular feature exceeded 30%, such as “previous obstetric history,” “presence of meconium,” and “O_Thyroid function blood,” we chose to exclude them from the analysis. For the remaining missing values in the features, we implemented an imputation strategy to enhance the usability of the data.

In terms of the choice of imputation method, we adopted a differentiated approach based on the type of feature. For numerical features, we used the median or mean for imputation, a method that reduces the impact of extreme values while preserving the distribution characteristics of the data. For categorical features, we used the k-nearest neighbors algorithm for imputation, which estimates the possible values of missing data by finding similar observations based on distance metrics.

Additionally, some categorical features with sparse or heterogeneous classifications were restructured using domain knowledge to improve statistical interpretability. For instance, antenatal medical factors were originally recorded in 64 different categories. These were clinically reviewed and consolidated into 7 meaningful groups: (1) mental health and neurological disorders, (2) genetic disorders, (3) surgical or operative conditions, (4) infectious and immune-related diseases, (5) obesity-related conditions, (6) chronic diseases, and (7) none.

Finally, to further meet the requirements of machine learning models, we performed 1-hot encoding on all categorical variables. This helps to avoid potential numerical encoding biases and ensures the effectiveness of model training.

### Feature Selection

**Figure 1.**
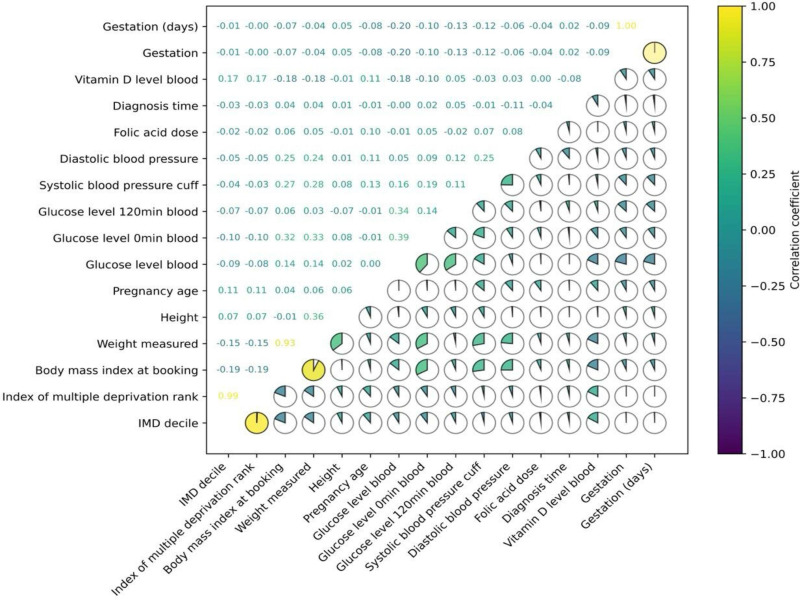
Pearson correlation matrix of key features for redundancy analysis. IMD: Index of Multiple Deprivation.

To mitigate the “curse of dimensionality” and enhance the practicality of model deployment in clinical settings, we removed redundant features. As shown in Figure 1, we used the Pearson correlation matrix to identify highly correlated variables. For continuous variables, feature selection was guided by the Pearson correlation coefficient. As the number of categorical variables was relatively small—primarily including “number of previous C-sections,” “obesity status,” and “antenatal medical factors”—all were retained in the model.

During the analysis, we identified several pairs of features with relatively high Pearson correlation coefficients, including “Index of Multiple Deprivation (IMD) decile” and “IMD rank,” “gestation (days)” and “gestation,” as well as “body mass index (BMI) at booking” and “weight measured.” Both deprivation-related variables reflect the socioeconomic status of pregnant women; however, given the broader range of values for “IMD decile” (1012 to 32,742), we considered it more statistically informative and retained this feature.

Given the direct association between gestational age and adverse pregnancy outcomes, gestational variables were excluded from the model to prevent potential confounding. Likewise, fetal birth weight—available only after delivery—was excluded to avoid outcome leakage. We recalculated BMI based on height and weight, as it offers a more comprehensive measure of maternal body composition. Consequently, the original “height” and “weight” variables were removed due to redundancy. Additionally, although “gestation (days),” “gestation,” and “Gravida” are strongly associated with adverse outcomes such as preterm birth and miscarriage, including them posed a risk of information leakage. Based on both statistical reasoning and clinical expert input, these features were excluded.

Lastly, “vitamin D level in blood” was removed due to concerns over data quality and consistency. Vitamin D is not a routine prenatal test, and the lack of standardization in test timing introduced considerable variability, limiting its reliability as a predictive feature.

### Missing Data

The final dataset included 10 continuous variables such as pregnancy age, blood glucose levels, and blood pressure. The categorical variables include obesity status, number of previous C-sections, and antenatal medical factors. Several continuous variables had missing values, with the highest rates observed in blood glucose levels (up to 23.1%) and blood pressure measurements (Multimedia Appendix 1).

### Model Construction

Based on our analysis results, we performed feature selection and data preprocessing and then constructed machine learning models. We used a variety of algorithms, including logistic regression, random forests, support vector machines (SVM), and extreme gradient boosting (XGBoost), to independently build classification prediction models. To further enhance the predictive power of the models, we used the Stacking ensemble learning method, taking the prediction results of these base models as input and training them with a meta-model (also known as a secondary model), thereby achieving superior overall predictive performance. In our study, we chose XGBoost as the meta-model. Considering that only 11.9% of the samples in the dataset manifested adverse pregnancy outcomes, indicating an imbalance in the dataset, we applied the adaptive synthetic sampling (ADASYN) algorithm to address this imbalance and improve the model’s generalization ability.

ADASYN is a machine learning algorithm used to address class imbalance in classification problems [17]. In imbalanced datasets, there is a large difference in the number of samples between different classes, which can result in poor classification performance of the model for minority classes. ADASYN balances the dataset by increasing the number of samples of the minority class, thus improving the model’s performance. Considering the imbalance between the alive group and the expired group and the impact on the accuracy of the prediction results, we applied the ADASYN method to oversample the training set, while the test set maintained the original sample ratio. To further validate the model’s robustness and performance in addressing class imbalance, we conducted supplementary experiments by introducing another widely used oversampling method—SMOTE (Synthetic Minority Oversampling Technique) for comparison. SMOTE generates synthetic minority class samples through interpolation in the feature space and has been extensively applied in imbalanced classification tasks. Compared with ADASYN, it is more comprehensive to evaluate the impact of different oversampling strategies on model performance, ensuring the selected method exhibits stronger robustness and generalization capability in practical applications. The overall data was split into a training set and a test set in an 8:2 ratio. To fine-tune the hyperparameters of each algorithm, we used grid search based on 5-fold cross-validation within the training set. The hyperparameters adjusted for each algorithm are as follows (Table 1).

**Table 1.** Hyperparameters adjusted for each algorithm.

Algorithm	Hyperparameters
Random forest	n_estimators: 10, 50, 100 max_features: auto, sqrt, log2 Bootstrap: true, false
Extreme Gradient Boosting	n_estimators: 20, 50, 100 learning_rate: 0.1, 0.2, 0.3 max_depth: 4, 6, 8 objective: binary: logistic subsample: 0.6, 0.8, 1
Logistic regression	C: 0.001, 0.01, 0.1, 1, 10, 100, 1000 Penalty: L1, L2
Support vector machine	C: 0.1, 1, 10, 100 Kernel: linear, rbf, poly Gamma: scale, auto

Based on the basic models obtained from training, we introduced the stacking strategy in our model fusion approach to more comprehensively explore the potential correlations between different basic models. We treated the outputs of each basic model as new features and further trained them using the XGBoost gradient boosting framework to construct a highly optimized stacking model with significant synergistic effects.

In clinical models, the interpretability of the model is crucial because it helps doctors and researchers understand the decision-making process of the model, thereby increasing trust in the model and promoting its application in clinical practice. SHAP is a popular machine learning interpretability tool that can quantify the contribution of each feature to the model’s prediction. In our study, we used the SHAP algorithm to perform an interpretability analysis on the gestational diabetes risk prediction model to increase the model’s transparency, helping doctors better understand the model’s decision-making process and make appropriate interventions when necessary.

### Ethical Considerations

This study is based on the publicly available GDM dataset. We included pregnant women who met specific diagnostic criteria, as defined in the dataset documentation. All protected health information has been deidentified to ensure privacy. The dataset is accessible , and its use complies with the associated public license and terms of use. Therefore, ethical approval and individual patient consent were not required for this study.

## Results

### Overview

**Figure 2.**
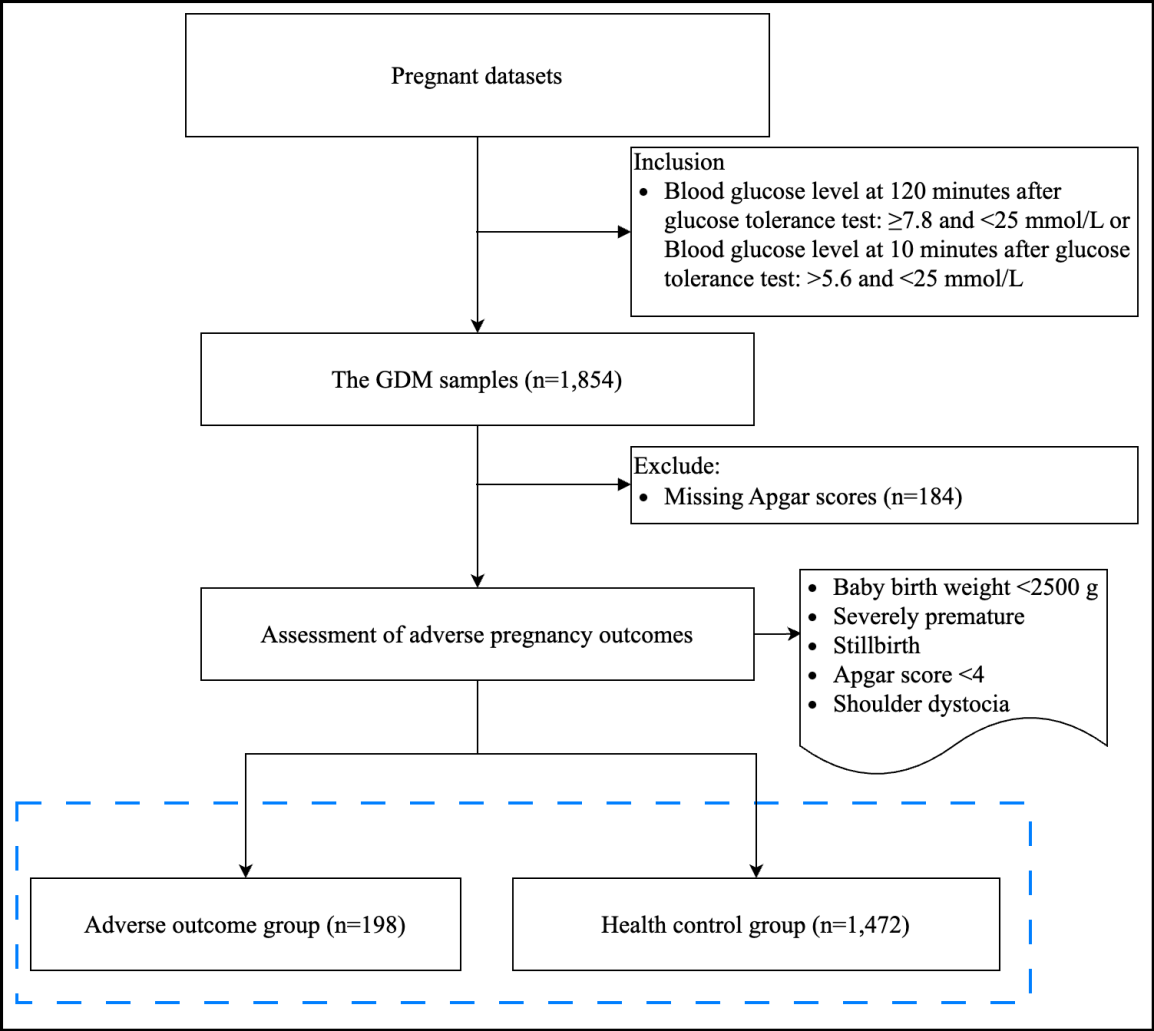
Flowchart of data inclusion and exclusion criteria. GDM: gestational diabetes mellitus.

A total of 1670 patients were selected from the GDM database for inclusion in this study. The data inclusion and exclusion diagram is shown in Figure 2. Among them, 200 patients had adverse pregnancy outcomes. Statistical analysis was conducted using SPSS (version 22.0; IBM Corp), while data cleaning, model construction, and performance evaluation were carried out using Python (Python Software Foundation). Continuous variables were expressed as median (IQR), while categorical data were presented as counts (percentages). The Mann-Whitney *U* test was used for analyzing continuous variables, and chi-square test was used to examine significant differences in categorical variables. The patient’s baseline is presented in Table 2.

**Table 2.** Comparison of maternal characteristics by outcome group. Statistical significance was defined as *P*<.05.

Variable	HC^a^	Adverse outcome	*P* value
IMD^b^ decile, median (IQR)	4.00 (3.00‐6.00)	4.00 (3.00‐6.00)	.25
Pregnancy age, median (IQR)	34.00 (30.00‐37.00)	35.00 (31.00‐38.00)	.04
Glucose level (blood), median (IQR)	4.70 (4.30‐5.30)	5.00 (4.45‐5.80)	<.001
Glucose level (0 min), median (IQR)	4.60 (4.20‐5.20)	4.70 (4.30‐5.65)	.002
Glucose level (120 min), median (IQR)	8.40 (8.00‐9.10)	8.50 (8.00‐9.50)	.02
Systolic blood pressure, median (IQR)	111.00 (104.00‐120.00)	115.00 (107.00‐125.00)	<.001
Diastolic blood pressure, median (IQR)	70.00 (63.00‐77.00)	72.00 (65.00‐79.25)	.01
Folic acid dose, median (IQR)	400.00 (0.00‐400.00)	400.00 (0.00‐400.00)	.81
Diagnosis time (weeks), median (IQR)	10.00 (10.00‐12.00)	10.00 (9.75‐12.00)	.82
BMI (kg/m²), median (IQR)	26.02 (22.81‐29.75)	26.51 (23.71‐30.05)	.02
Obese? n (%)			.30
No	908 (74.49)	324 (71.84)	
Yes	311 (25.51)	127 (28.16)	
No_of_previous_csections, n (%)			.69
0	1003 (82.28)	369 (81.82)	
1	187 (15.34)	72 (15.96)	
2	26 (2.13)	7 (1.55)	
3	2 (0.16)	2 (0.44)	
4	1 (0.08)	1 (0.22)	
Antenatal medical factors, n (%)			.10
Chronic diseases	117 (9.60)	50 (11.09)	
Genetic disorders	5 (0.41)	0 (0)	
Infectious and immune-related diseases	27 (2.21)	12 (2.66)	
Mental health and neurological disorders	47 (3.86)	30 (6.65)	
Obesity-related conditions	2 (0.16)	2 (0.44)	
Surgical or operative conditions	110 (9.02)	43 (9.53)	
None	911 (74.73)	314 (69.62)	

a HC: healthy control.

b IMD: Index of Multiple Deprivation.

In this study, we evaluated 5 different machine learning models: random forest, XGBoost, logistic regression, SVM, and a stacking ensemble model. To address class imbalance in the training data, we applied oversampling techniques such as ADASYN before model training. However, to preserve the clinical authenticity and real-world applicability of the evaluation, no oversampling was applied to the test set. A comprehensive evaluation was conducted using multiple performance metrics, including sensitivity, specificity, positive predictive value (PPV), negative predictive value (NPV), positive likelihood ratio (PLR), negative likelihood ratio (NLR), and overall accuracy. The results obtained on the test set are summarized in Table 3 below.

**Table 3.** Comparative performance metrics of machine learning models for predicting GDM^a^-related adverse outcomes.

Model	SEN^b^	SPE^c^	PPV^d^	NPV^e^	PLR^f^	NLR^g^	Accuracy	*F*_1_-score
Random forest	0.600	0.926	0.750	0.863	8.133	0.432	0.838	0.667
XGBoost^h^	0.567	0.955	0.823	0.857	12.570	0.454	0.850	0.671
Logistic regression	0.478	0.721	0.387	0.789	1.714	0.724	0.656	0.428
SVM^i^	0.633	0.828	0.576	0.860	3.679	0.443	0.775	0.603
Stacking	0.578	0.959	0.839	0.860	14.098	0.440	0.856	0.684

a GDM: gestational diabetes mellitus.

b SEN: sensitivity.

c SPE: specificity.

d PPV: positive predictive value.

e NPV: negative predictive value.

f PLR: positive likelihood ratio.

g NLR: negative likelihood ratio.

h XGBoost: extreme gradient boosting.

i SVM: support vector machine.

The results showed that the stacking model outperformed the individual models across several key metrics. Through cross-validation, we evaluated accuracy, recall, precision, and *F*_1_-score. The results indicated that the stacking model excelled in specificity (0.959), PPV (0.839), and PLR (14.098). Its sensitivity (0.578) and NPV (0.860) were also strong, with a low NLR (0.440) and an overall accuracy of 0.856, demonstrating the effectiveness of the model fusion approach.

**Figure 3.**
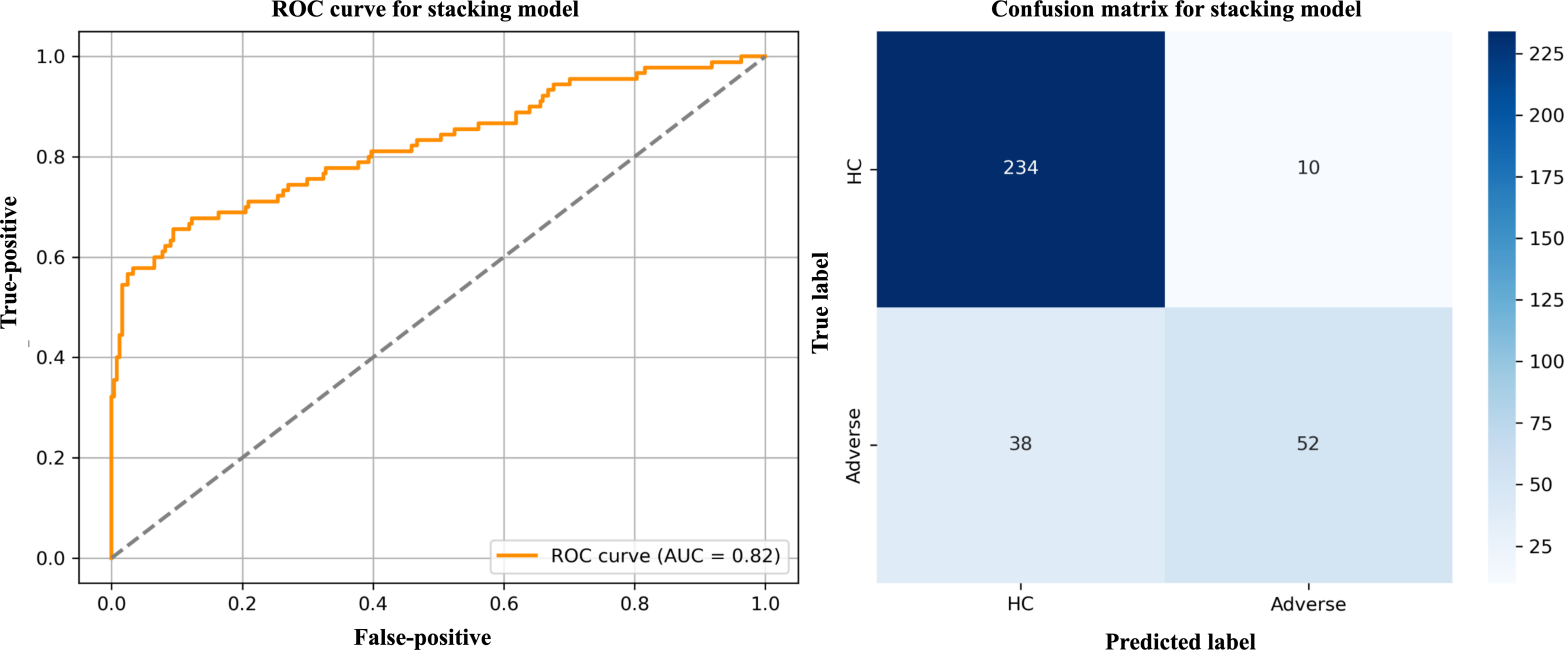
ROC curve and confusion matrix of the stacking model for predicting GDM-related adverse outcomes. AUC: area under the curve; GDM: gestational diabetes mellitus; HC: healthy control; ROC: receiver operating characteristic.

A total of 334 samples were contained in the test set, including 90 cases with adverse pregnancy outcomes (positive class) and 244 cases with favorable outcomes (negative class). The positive-to-negative ratio is approximately 1:2.7, indicating a moderately imbalanced distribution. Based on this dataset, the model demonstrated a satisfactory performance in identifying adverse outcomes, achieving an area under the receiver operating characteristic curve of 0.82. This suggests that the model exhibits high overall discriminative ability and can effectively differentiate individuals with distinct pregnancy outcomes. The receiver operating characteristic curve and confusion matrix for the stacking model are presented in Figure 3.

To further investigate the effect of different oversampling techniques on model performance, we conducted a comparative experiment using both ADASYN and SMOTE, focusing on the stacking model, which showed the best overall performance in our main evaluation. The results are presented in Table 4.

**Table 4.** Performance comparison of ADASYN^a^ and SMOTE^b^.

Oversampling method	SEN^c^	SPE^d^	PPV^e^	NPV^f^	PLR^g^	NLR^h^	Accuracy	*F*_1_-score
ADASYN (stacking)	0.578	0.959	0.839	0.860	14.098	0.440	0.856	0.684
SMOTE (stacking)	0.266	0.910	0.477	0.800	2.947	0.807	0.728	0.341
/	0.658	0.506	0.292	0.827	1.332	0.676	0.542	0.405

a ADASYN: adaptive synthetic sampling.

b SMOTE: Synthetic Minority Oversampling Technique.

c SEN: sensitivity.

d SPE: specificity.

e PPV: positive predictive value.

f NPV: negative predictive value.

g PLR: positive likelihood ratio.

h NLR: negative likelihood ratio.

As shown, the stacking model trained with ADASYN achieved superior performance across most metrics, particularly in terms of sensitivity (0.578), PPV (0.839), and *F*_1_-score (0.684), suggesting better identification of high-risk cases and overall balance between precision and recall. In contrast, the model trained with SMOTE exhibited a much lower sensitivity (0.266) and *F*_1_-score (0.341), indicating its limited ability to correctly identify minority class samples in this setting. We also report the performance of the stacking model trained without any oversampling as a reference, which demonstrated relatively higher sensitivity (0.658) but at the cost of much lower precision (PPV 0.292) and specificity (0.506), further confirming the advantage of using ADASYN in managing class imbalance.

**Figure 4.**
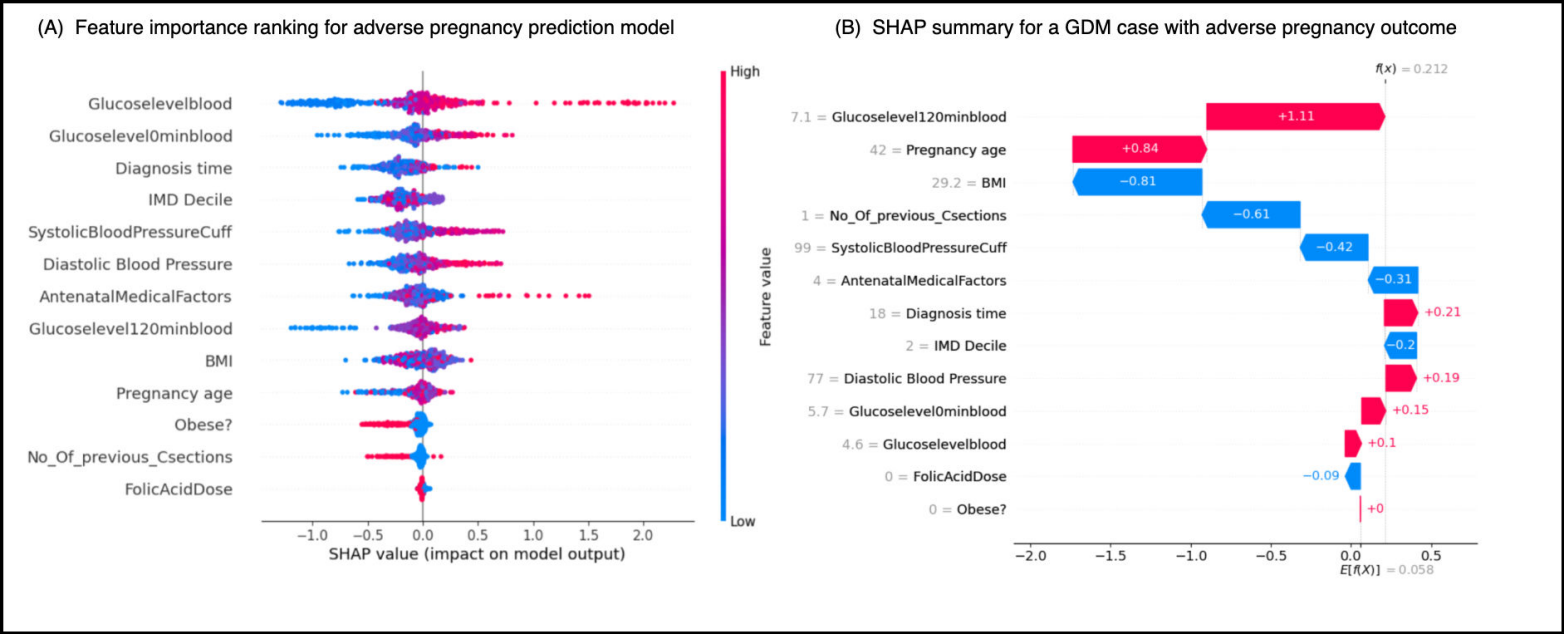
SHAP-based visualization of a machine learning model for predicting adverse pregnancy outcomes in patients with GDM. (A) Summary plot of SHAP values for all features, showing their impact and direction of effect on model output across all patients. (B) Local explanation for a single GDM case, highlighting how individual features contributed to the prediction. GDM: gestational diabetes mellitus; IMD: Index of Multiple Deprivation; SHAP: Shapley additive explanations.

**Figure 5.**
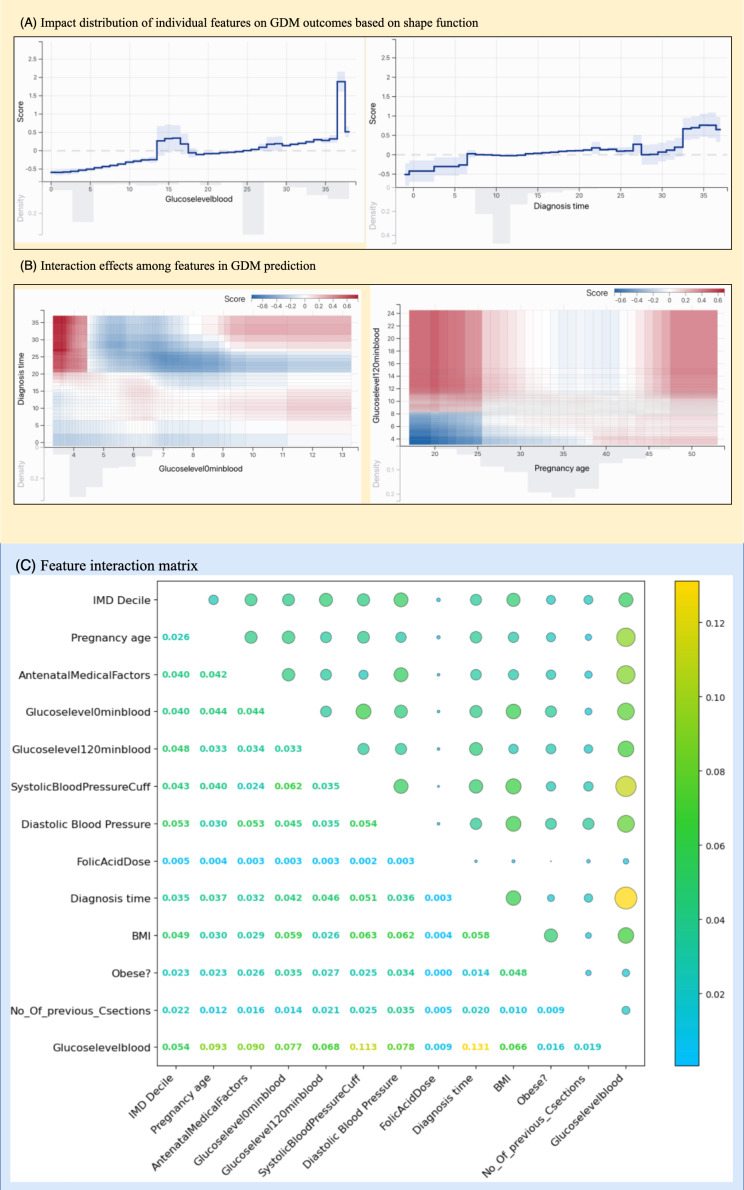
SHAP-based interpretation of feature effects and interactions in the prediction model. GDM: gestational diabetes mellitus; IMD: Index of Multiple Deprivation; SHAP: Shapley additive explanations.

Additionally, by using the SHAP algorithm, we conducted an in-depth analysis of the model’s interpretability. The SHAP values revealed that clinical characteristics significantly impact the prediction of gestational diabetes risk, with specific results as follows. Through the calculation and analysis of SHAP values, we were able to uncover the contribution of each feature to the GDM outcome, thereby gaining a more comprehensive understanding of the risk factors for GDM. This interpretable analysis not only aids in validating the robustness of the model but also provides valuable references for subsequent clinical decision-making. Based on the SHAP algorithm, we were able to perform precise visual analyses for individual patients, as shown in Figure 4, which includes a global summary of feature importance across all patients (Figure 5A) and a local explanation for an individual prediction (Figure 5B).

For each feature, we used the shape function to display the distribution of its different values and their impact on the outcome. The influence of individual features was shown in Figure 5A, which demonstrates the specific impact of changes in a single variable’s values on the model’s predicted risk, revealing nonlinear relationships. Additionally, we used visualization techniques to illustrate the interaction values between different features, as shown in Figure 5B. These analyses will enhance our ability to conduct clinical assessments more effectively.

We further conducted feature interaction analysis to explore the contribution of different feature combinations in predicting adverse pregnancy outcomes in GDM. As shown in Figure 5C, the plot illustrates the overall importance of each feature in the model and its direction of influence, with each dot representing the SHAP value of an individual patient. The features are ranked by their mean absolute SHAP values, reflecting their average impact magnitude on the model’s output. These results also highlight the significance of key variables and their interactions in clinical risk prediction.

### External Validation

Based on the integrated model we ultimately obtained, we collected data from some patients with GDM at Jinniu Maternal and Child Health Hospital (from January 2021 to March 2024) through telephone follow-up, focusing on collecting data from patients with adverse pregnancy outcomes. Due to issues such as patient cooperation and the tightness of related human resources, the number of external validation data is relatively small (159 cases, with a positive to negative ratio of 27:132). We used the stacking model for validation, which had a final accuracy of 0.836 and an area under the receiver operating characteristic curve value of 0.669, showing a significant decline compared to the test set. The results are shown in Multimedia Appendix 2.

## Discussion

### Principal Findings

According to the latest Global Diabetes Atlas (9th edition) published by the International Diabetes Federation, the number of patients with GDM worldwide is increasing, with approximately 20.4 (15.8%) million women enduring hyperglycemia; among them, around 17.1 (83.6%) million cases are due to GDM [18]. Pregnant women affected by GDM face an elevated risk of adverse pregnancy outcomes, which can pose significant threats to both the mother and the infant. Therefore, predicting adverse pregnancy outcomes in the early stages of pregnancy and providing timely feedback and effective clinical management are particularly important.

The traditional scoring criteria for adverse pregnancy outcomes currently used in clinical practice, such as the IADPSG (International Association of Diabetes and Pregnancy Study Groups) and the NICE (National Institute for Health and Care Excellence) Diagnostic Criteria, exhibit varying levels of predictive efficacy and are prone to regional biases, making it difficult to achieve a consensus on predictive outcomes [19,20]. Additionally, individual differences, such as physiological variations, hormonal changes, genetics, and dietary structures, also affect the estimated prevalence rates of GDM. However, current clinical strategies for gestational diabetes typically use a one-size-fits-all approach to blood glucose management. Therefore, more comprehensive regular monitoring and earlier preventive and intervention measures should be implemented to reduce the potential for adverse outcomes.

In this study, we compared 5 machine learning models, random forest, XGBoost, logistic regression, SVM, and stacking, to assess their performance in predicting outcomes related to GDM. The results indicated that the stacking model outperformed the other models across multiple key performance metrics, particularly excelling in specificity (0.959), PPV (0.839), and PLR (14.098). Additionally, the stacking model demonstrated good performance in sensitivity (0.578) and NPV (0.860), with a low NLR (0.44) and overall accuracy (0.856), further proving the significant advantage of model ensemble methods in enhancing predictive performance. The receiver operating characteristic curve and confusion matrix analysis also supported this conclusion, emphasizing the effectiveness of the stacking model in predicting adverse pregnancy outcomes in GDM. However, during external validation, when using domestic patient data, the model’s performance significantly declined. This phenomenon may be related to the inconsistent distribution of dataset characteristics, such as differences in demographic characteristics, lifestyle habits, and medical environments between domestic and international datasets. These differences may lead to poor model adaptation when facing new data distributions. Future research should further explore the performance of these models on larger and more diverse datasets to verify their generalization and stability. At the same time, it is recommended to integrate clinical context by introducing more features related to gestational diabetes for analysis, such as real-time vital signs of pregnant women, genetic phenotypes, and other relevant indicators, to enhance the model’s predictive power and clinical application value.

SHAP helps clinicians understand and interpret complex machine learning models. We noticed that adverse pregnancy outcomes have a significant association with gestational age. Previous studies have shown that adverse pregnancy outcomes are directly related to blood glucose control during the perinatal period of gestational diabetes [21]. Our results indicate that gestational diabetes with a shorter duration during pregnancy is associated with poorer pregnancy outcomes, which may be related to the early detection of hyperglycemia leading to preterm birth. Therefore, these findings serve as an important alert for clinical practice, suggesting that blood glucose monitoring should be initiated earlier in pregnancy, considering the patient’s condition, to conduct earlier glucose monitoring for different individuals. Current clinical guidelines recommend screening for gestational diabetes between 24 and 28 weeks of pregnancy, and screening for and treating gestational diabetes during this period can significantly reduce the risk of adverse pregnancy outcomes [4,22]. Meanwhile, a large cohort study found that women diagnosed with GDM before 12 weeks of pregnancy had worse pregnancy outcomes compared to those diagnosed between 24‐28 weeks [23]; the research by Mustafa et al [7] also found that women with early onset (<24 weeks) GDM had higher BMI, significantly increased demand for insulin, and more adverse pregnancy outcomes. Therefore, in addition to recommending universal screening at 24‐28 weeks, identifying and treating GDM at an earlier stage could reduce the potential perinatal or long-term adverse effects on the mother and the offspring. These findings emphasize the importance of identifying GDM earlier than current practices and exploring other interventions that might improve outcomes beyond blood glucose control.

Pregnant women with GDM experience insulin resistance, as previously reported in studies, which is associated with preterm birth, and preterm newborns are at a higher risk of being admitted to the neonatal intensive care unit or special care baby unit [24]. Due to pregnant women with high blood glucose levels, adverse pregnancy outcomes such as preterm birth, large-for-gestational-age infants, and neonatal hypoglycemia often require care in a neonatal intensive care unit. Excess glucose in the mother’s circulation is transferred across the placenta to provide the fetus with energy substrates. The fetus’s response to the excess substrates is to produce more insulin, leading to fetal hyperinsulinemia, which can result in a series of consequences, particularly rapid fetal growth [25]. In pregnancies with GDM, fetal insulin binds to insulin-like growth factor 1 receptors, exerting growth hormone-like effects and serving as a key factor in promoting fetal growth [26]. It is common for children born to women with GDM to be large at birth. Extensive research on this population has shown that the increased birth weight of large-for-gestational-age infants is due to an increase in fat mass, not muscle mass [27,28]. The impact of GDM on both mother and infant does not end at delivery. Newborns admitted to the neonatal intensive care unit or special care baby unit due to maternal gestational diabetes are often seen with neonatal hypoglycemia, which may occur in 10% of healthy term infants, mainly within the first 24 to 48 hours after birth [29]. The high glucose consumption of the brain, coupled with the increased ratio of brain to body mass in newborns compared to adults, increases the demand for glucose in newborns and may expose them to the risk of neurological damage and adverse outcomes.

Pregnancy hypertension is a common complication of pregnancy and a leading cause of morbidity and mortality in pregnant women and newborns, accounting for about 14% of maternal deaths worldwide [30,31]. Both pregnancy-induced hypertension and pre-eclampsia are associated with adverse maternal and fetal outcomes, including an increased risk of future maternal cardiovascular diseases, and there is approximately a 17% chance of pregnancy-induced hypertension progressing to pre-eclampsia [32]. As many women are asymptomatic at the time of diagnosis, those who do not receive prenatal care may present with more advanced hypertension-related diseases, such as eclampsia, which is associated with higher risks of morbidity and mortality [33]. In correlation analyses, GDM is accompanied by increased blood pressure in late pregnancy. GDM is a known risk factor for stillbirth, fetal macrosomia, fetal structural abnormalities, preterm birth, and pregnancy-induced hypertension diseases. GDM is characterized by significantly increased concentrations of inflammatory molecules and the imbalanced expression of genes encoding inflammatory mediators in the placenta, which together lead to the disruption of vascular, metabolic, and inflammatory processes. Over time, this state of hyperglycemia may be associated with the occurrence of pregnancy-induced hypertension diseases and their related complexities. Additionally, diabetes and hypertension often coexist, sharing various risk factors and disease etiologies, including genetics, obesity, insulin resistance, and inflammation [34]. Therefore, the management of GDM should not only focus on blood glucose control but also consider the potential risks associated with pregnancy-induced hypertension diseases. In GDM, oxidative stress plays a role in the pathogenesis of the disease, as excessive secretion of insulin during pregnancy leads to the production of lipid peroxidation factors, which also mask the secretion of antioxidants, making reactive oxygen species abundant at the cellular level. In pre-eclampsia and gestational hypertension, oxidative stress leads to inadequate placental perfusion, resulting in a hypoxic placenta, which generally triggers a systemic maternal inflammatory response [35-37].

The adverse pregnancy risks associated with GDM are determined by the complex interplay of multiple biomedical, behavioral, and environmental factors‚ rather than a single factor. Therefore, to investigate the contribution of feature interaction to the prognostic risks of GDM, we conducted multifeature interaction analyses. The feature interaction matrix clearly shows that blood glucose levels and time of diagnosis exhibit the highest degree of aggregation, followed by blood pressure. Additionally, gestational age and antenatal medical factors also demonstrate relatively strong clustering patterns. As previously discussed, individualized diagnosis and treatment should be tailored to different individuals. For those with a genetic predisposition to diabetes, earlier diagnosis during pregnancy should be implemented to achieve more timely blood glucose control. Hypertensive disorders in pregnancy and GDM are among the most common metabolic complications during gestation, with prevalence rates having risen significantly over the past decade, now affecting approximately 12%‐18% of all pregnancies [38]. Pregnant women with hyperglycemia generally exhibit a higher overall incidence of requiring insulin therapy, which increases with maternal age, and women over 35 years old have a significantly higher probability of undergoing cesarean delivery [39]. Genes are the most important factor in Antenatal Medical Factors. More recent genome-wide association studies focusing on maternal metabolism during pregnancy have revealed overlaps between genes associated with metabolic traits in pregnant and nonpregnant populations, as well as some genes that appear to be uniquely relevant during gestation. Additionally, reduced gene expression has been observed in transcription factors involved in lipid metabolism, such as LXRa, PPARa, PPARd, PPARg, RXRa, and SREBP1c. These findings indicate the importance of maternal oversupply of substrates in determining neonatal fat accumulation in pregnancies complicated by obesity and GDM [40].

### Limitations

This study has several limitations. As a single-center retrospective study with a relatively small sample size, the generalizability of the findings may be limited. Although multiple machine learning models were used, the features were primarily derived from structured clinical data, lacking unstructured information such as imaging findings and physician notes. And the external validation dataset also limited our research; the model’s performance notably declined on domestic datasets, suggesting potential overfitting or insufficient adaptability to different populations. Although we use SHAP for interpretability analysis, the model’s clinical transparency remains constrained due to the complex and nonlinear relationships between features and outcomes, which may affect the interpretability of clinical decision-making.

### Conclusion

In conclusion, our study highlights the growing prevalence of GDM and its significant association with adverse pregnancy outcomes, including preterm birth, large-for-gestational-age infants, and neonatal hypoglycemia. Through the evaluation of 5 machine learning models, we found that the stacking model exhibited superior performance in predicting adverse outcomes, demonstrating the potential of machine learning to enhance clinical decision-making in managing GDM. The findings emphasize the importance of early identification and personalized management of GDM to mitigate the risks to both mother and infant. Moreover, incorporating additional clinical features, such as real-time maternal vital signs and genetic phenotypes, may further enhance the predictive accuracy and clinical applicability of these models. Future studies should aim to validate these findings across more diverse datasets to ensure the generalizability and robustness of the models, paving the way for more precise and effective interventions in GDM management.

## Supplementary material

10.2196/71539 Multimedia Appendix 1 Missing value rates of variables in the final dataset.

10.2196/71539 Multimedia Appendix 2 External validation outcomes for the stacking model in patients with gestational diabetes mellitus.

## Abbreviations

ADASYN: adaptive synthetic sampling
GDM: gestational diabetes mellitus
IADPSG: International Association of Diabetes and Pregnancy Study Groups
IMD: Index of Multiple Deprivation
NICE: National Institute for Health and Care Excellence
NLR: negative likelihood ratio
NPV: negative predictive value
OGTT: oral glucose tolerance test
PLR: positive likelihood ratio
PPV: positive predictive value
SHAP: Shapley additive explanation
SMOTE: Synthetic Minority Oversampling Technique
SVM: support vector machine
XGBoost: extreme gradient boosting
