# Supplementary figures and images for: Interpretable Machine Learning for Predicting Adverse Pregnancy Outcomes in Gestational Diabetes: Retrospective Cohort Study
Source: JMIR Med Inform. 2025 Sep 16;13:e71539. doi: 10.2196/71539 (PMC12441465; doi:10.2196/71539)

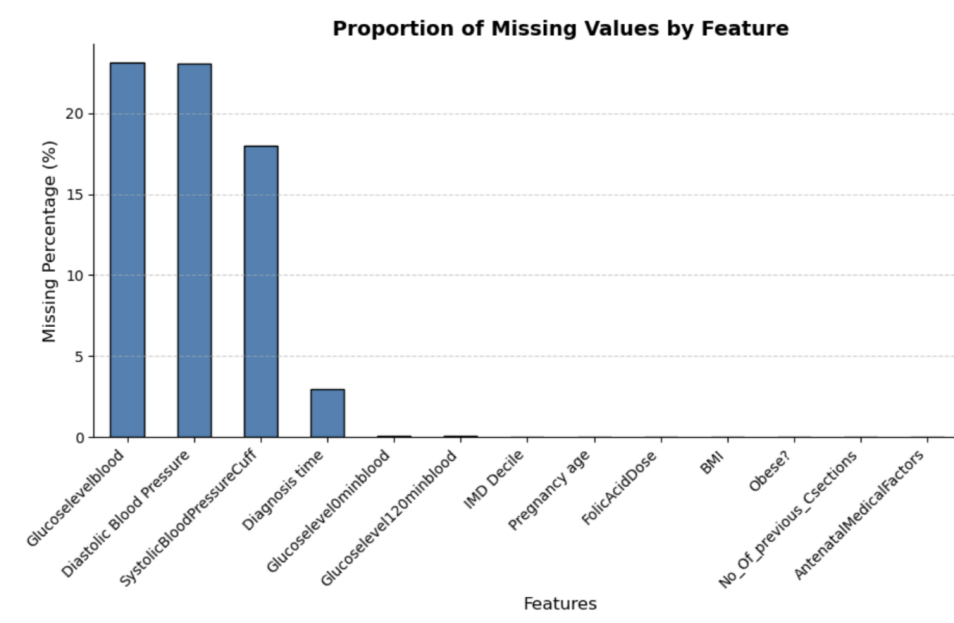

Supplement: Multimedia Appendix 1 [file medinform-v13-e71539-s001.png]

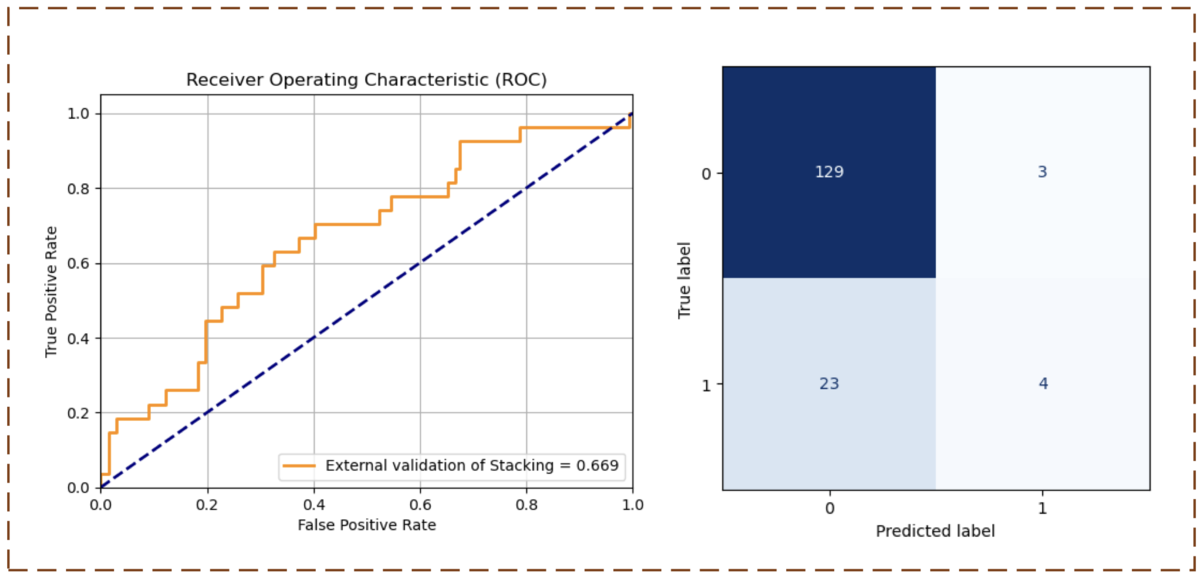

Supplement: Multimedia Appendix 2 [file medinform-v13-e71539-s002.png]
